# Murine CD4^+^CD25^-^ cells activated *in vitro* with PMA/ionomycin and anti-CD3 acquire regulatory function and ameliorate experimental colitis *in vivo*

**DOI:** 10.1186/1471-230X-12-172

**Published:** 2012-12-03

**Authors:** Anna Majowicz, Sander van der Marel, Anje A te Velde, Sybren L Meijer, Harald Petry, Sander J van Deventer, Valerie Ferreira

**Affiliations:** 1 Research and Development, uniQure B.V., Meibergdreef 61, 1105 BA, Amsterdam, The Netherlands; 2 Department of Gastroenterology & Hepatology, Leiden University Medical Center, Albinusdreef 2, 2333 ZA, Leiden, The Netherlands; 3 Tytgat Institute for Liver and Intestinal Diseases, Meibergdreef 69-71, 1105 BK, Amsterdam, The Netherlands; 4 Department of Pathology, Academic Medical Center, Meibergdreef 9, 1105 AZ, Amsterdam, The Netherlands

**Keywords:** IBD, CD45RB transfer, Treg, PMA/ionomycin

## Abstract

**Background:**

Induced regulatory T (iTreg) lymphocytes show promise for application in the treatment of allergic, autoimmune and inflammatory disorders. iTreg cells demonstrate advantages over natural Treg (nTreg) cells in terms of increased number of starting population and greater potential to proliferate. Different activation methods to generate iTreg cells result in iTreg cells that are heterogeneous in phenotype and mechanisms of suppression. Therefore it is of interest to explore new techniques to generate iTreg cells and to determine their physiological relevance.

**Methods:**

Using phorbol myristate acetate (PMA)/ionomycin and anti-CD3 activation of CD4^+^CD25^-^ cells we generated *in vitro* functional CD4^+^CD25^+^ iTreg (TregPMA) cells. Functionality of the generated TregPMA cells was tested *in vivo* in a mouse model of inflammatory bowel disease (IBD) - CD45RB transfer colitis model.

**Results:**

TregPMA cells expressed regulatory markers and proved to ameliorate the disease phenotype in murine CD45RB transfer colitis model. The body weight loss and disease activity scores for TregPMA treated mice were reduced when compared to diseased control group. Histological assessment of colon sections confirmed amelioration of the disease phenotype. Additionally, cytokine analysis showed decreased levels of proinflammatory colonic and plasma IL-6, colonic IL-1 β and higher levels of colonic IL-17 when compared to diseased control group.

**Conclusions:**

This study identifies a new method to generate *in vitro* iTreg cells (TregPMA cells) which physiological efficacy has been demonstrated *in vivo*.

## Background

T regulatory (Treg) lymphocytes are a cellular component of the immune system responsible for suppressing immune responses of effector cells. As a result of their ability to control immune responses and to sustain systemic immune balance, they have the potential to prevent allergic, autoimmune and inflammatory disorders [1-10] as well as to be an adjuvant therapy for chronic and acute graft versus host disease [11].

The safety profile of Treg cells has been established in phase I clinical trials [11,12] demonstrating that Treg cells are a suitable candidate for therapeutic purposes. However a major limitation to the clinical use of natural Treg (nTreg) cells is their low availability as they represent only a small percentage of the peripheral circulating CD4^+^ T cell population. In order to overcome this issue, several groups have developed methods to expand nTreg cells *in vitro* when keeping their functionality. Generally the technologies are complex, time-consuming and the plasticity of nTreg cell lineage in artificial environment during *ex vivo* expansion can lead to loss of their suppressive activity [13]. Furthermore their stage of differentiation makes their expansion *in vitro* a difficult process [14].

*In vitro* induced T regulatory (iTreg) cells represent a good alternative to nTreg cells since they have been reported to have similar functionality in *in vivo* setup. Additionally iTreg cells show advantages over nTreg cells in terms of increased number of starting population and greater potential to proliferate [15]. Therefore it is of importance to investigate and explore new approaches to generate iTreg cells.

Based on the activating and stimulating properties of phorbol myristate acetate (PMA)/ionomycin and anti-CD3 on T cells [16,17] we developed a new method to generate iTreg cells *in vitro*, which we refer to as ‘TregPMA’ cells. The functionality of the TregPMA cells was assessed *in vivo* in a mouse model of experimental colitis.

## Methods

### Mice

BALB/C and C.B.-17 SCID mice (8–10 weeks) were obtained from Harlan and maintained in specific pathogen-free conditions. Mouse experiments were approved by the local animal welfare committee (University of Amsterdam).

### Generation of regulatory T lymphocytes (Treg PMA cells)

Splenocytes were isolated from BALB/C mice. CD4^+^ CD25^-^ T cells were isolated from splenocytes by means of negative selection using the mouse “CD4^+^ T Cell Isolation Kit” followed by “CD25 MicroBead Kit” (Miltenyi Biotec). Cells were seeded at day 0 at 1x10^5^/well into anti-CD3e (0.5 μg/well, clone 145-2C11, eBioscience) coated 96-well flat bottom plates (Costar) and cultured in X-VIVO 15 medium (Lonza) at 37°C in a 5% CO_2_ incubator. On day 1, cells were activated with PMA (10 ng/ml) and ionomycin (250 ng/ml) and at day 2, 3 and 4 supplemented with 20 U/well of IL-2 (eBioscience). On day 5, prior to use, cells were harvested and treated with “Dead Cell Removal Kit” (Miltenyi Biotec).

### Flow cytometry analysis

Cells were analyzed using flow cytometry (FACSCalibur, BD Biosciences). For Treg cell staining the mouse “Regulatory T cell Staining Kit” was used which consisted of anti-mouse CD4 FITC (clone RM4-5), anti-mouse CD25 APC (clone PC61.5) and anti-mouse/rat Foxp3 PE (clone FJK16). Additional monoclonal anti-mouse flow cytometry antibodies used in this study were as follows: anti-mouse CD152 (CTLA-4) APC (clone UC10-4B9), anti-mouse GARP PE (clone YGIC86), anti-mouse/rat CD278 (ICOS) FITC (clone C398.4A) and anti-mouse CD134 (OX-40) APC (clone OX86). All antibodies were obtained from eBioscience.

### Induction of CD45RB transfer colitis model and treatment with TregPMA cells

Chronic colitis was induced in C.B.-17 SCID mice by intraperitoneal (IP) injection of CD45RB^high^ cells (4x 10^5^) isolated from normal BALB/C mice splenocytes (positive control group). Mice that received CD45RB^high^ cells in combination with CD45RB^low^ cells (2x10^5^) were protected from disease development (negative control group). The treatment group received CD45RB^high^ cells in combination with *in vitro* generated TregPMA cells (TregPMA cell treated group). The amount of TregPMA cells injected per mouse was 1.2x10^6^.

### Monitoring development of colitis

The primary read-out to assess the development of colitis was the body weight loss. Mice were weighed three times a week. Body weight loss was determined by percentage of weight loss from base line body weight.

After sacrifice, colon was excised and longitudinally divided into 2 parts both of which were rolled up: one was used for preparation of paraffin embedded samples while the other was snap frozen in liquid nitrogen. Disease activity index (DAI) was calculated by combining the scores applied to weight loss (0: <1%; 1: 1-5%; 2: 5-10%; 3: 10-15%; 4: >15%), stool consistency at sacrifice (0: normal; 1: loose droppings; 2: loose stools, colon filled with feces; 3: loose stool, feces only near caecum, 4: empty bowel) and rectal bleeding (0: negative; 2: positive; 3: gross bleeding) divided by three.

### Histology

The half divided colon tissue was fixed in 4% paraformaldehyde and embedded in paraffin. The colon tissue was cut in 5 μm sections and stained with hematoxylin and eosin for histological scoring. An experienced pathologist blinded to experiment inspected microscopically all sections and graded them on a scale 0 to 4 looking at: mononuclear and polymorphonuclear infiltrate, goblet cell depletion, crypt loss, epithelial hyperplasia, presence of ulcerations and manifestations of crypt abscesses.

### Cytokine analysis

Frozen colon tissue was crushed with the use of CryoPrep™ System (Covaris) and resuspended in ice-cold PBS (pH 7.2) containing complete protease inhibitor (Roche). Homogenates were then centrifuged at 15000 x g for 5 min at 4°C and the supernatants were stored at −80°C until the cytokine assay. Prior to cytokine analysis total concentration of the protein in supernatants was determined with the use of Bradford assay (Biorad). Colonic tissue and plasma levels of IL-1β, IL-6, IL-10, IL-17 and TNF-α were analyzed using a mouse cytokine magnetic bead-based multiplex assay (Biorad) according to the manufacturer’s instructions. Additionally TGF-β levels were analyzed with Bio-Plex Pro TGF-β assay (Biorad) according to manufacturer’s instructions.

### Statistical analysis

Data were analyzed statistically and graphed using Prism 5.0 (GraphPad Software). Changes in mice weekly body weight, body weight at sacrifice, disease activity index (DAI), histological score and cytokine levels are shown as mean ± standard deviation (SD) and analyzed by one-way ANOVA, followed by Bonferroni’s Multiple Comparison Test. *P < 0.05, **P < 0.01, ***P < 0.001, ****P < 0.0001.

## Results

### CD4^+^CD25^-^ T cells acquire a regulatory T cell phenotype through PMA/ionomycin/anti-CD3 mediated activation

**Figure 1:**
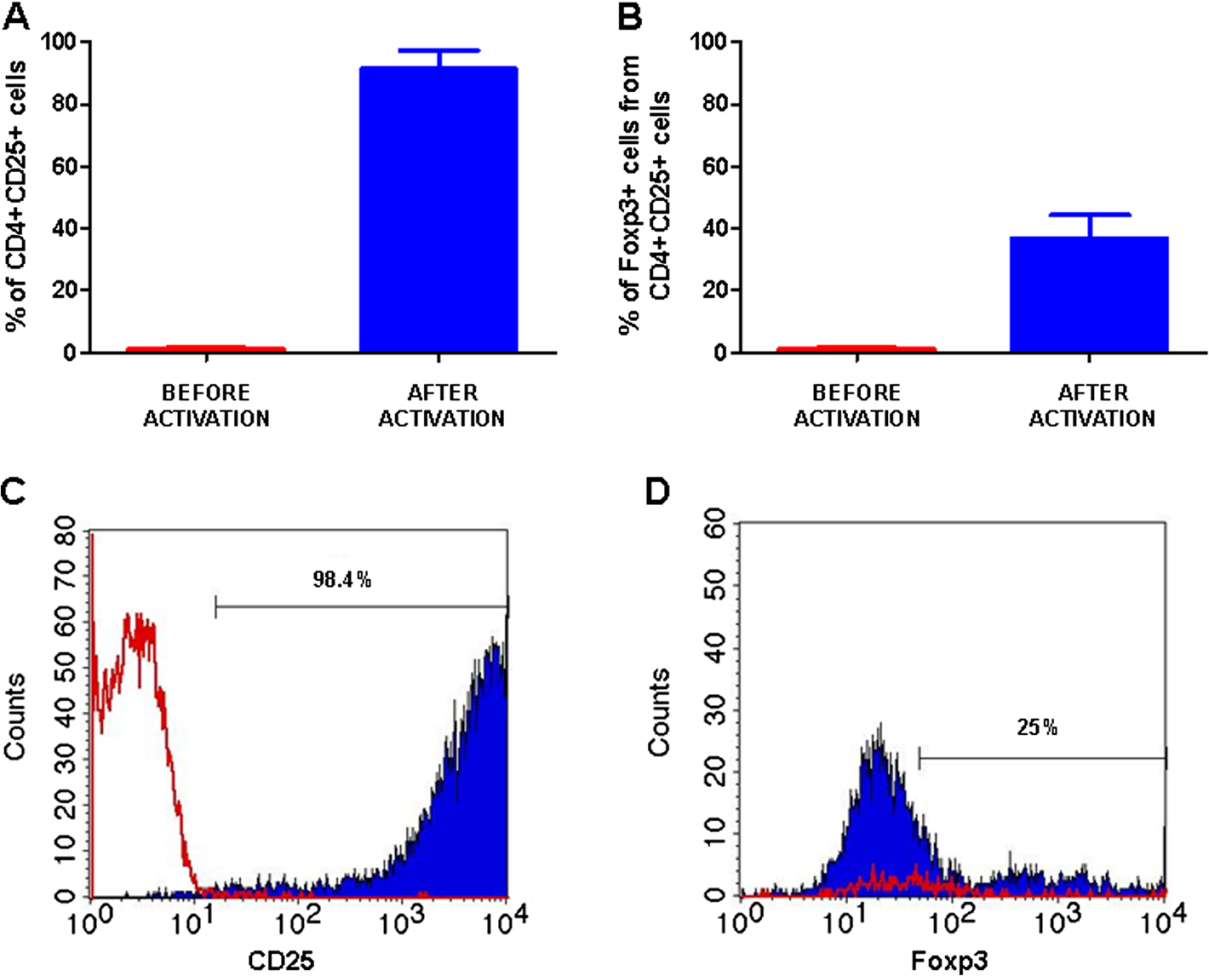
**CD4**^**+**^**CD25**^**-**^**cells acquire regulatory phenotype-CD4**^**+**^**CD25**^**high**^**Foxp3**^**+**^**.** After activation with PMA/ionomycin/anti-CD3 and 5 days in culture, the CD4^+^CD25^-^ T cells were analyzed by flow cytometry and 85.26% to 91.85% (mean: 91.4 ± 5.9%, n = 3) of the cells were shown to co-express CD4 and CD25 marker (**A**) and mean of 36.86 ± 7.46% of the cells acquire the classical regulatory phenotype- CD4^+^CD25^high^Foxp3^+^ (**B**). Three independent experiments were performed (**A**, **B**). Representative overlays of CD25 histograms (**C**) and Foxp3 (**D**) before (in red) and after (in blue) PMA/ionomycin/anti-CD3 activation and 5 days in culture. One representative experiment is shown. Foxp3 histogram was gated on CD4^+^CD25^+^ cell population (**D**).

In order to generate regulatory T cells, CD4^+^CD25^-^ cells from BALB/C mice were activated overnight on 96-well plate coated with anti-CD3. Subsequently PMA/ionomycin and IL-2 were added to the cell culture. After 5 days in culture, the cells were analyzed for their phenotype by flow cytometry. Between 85.26% and 91.85% of the cultured cells were shown to up-regulate and co-express the CD4 and CD25 markers (mean 91.4 ± 5.9%, n = 3, see: Figure 1A, C). The absence of PMA/ionomycin activation step on CD4^+^CD25^-^ cells resulted in significant decrease in cell viability (data not shown) which confirms the necessity of additional activation with PMA/ionomycin.

**Figure 2:**
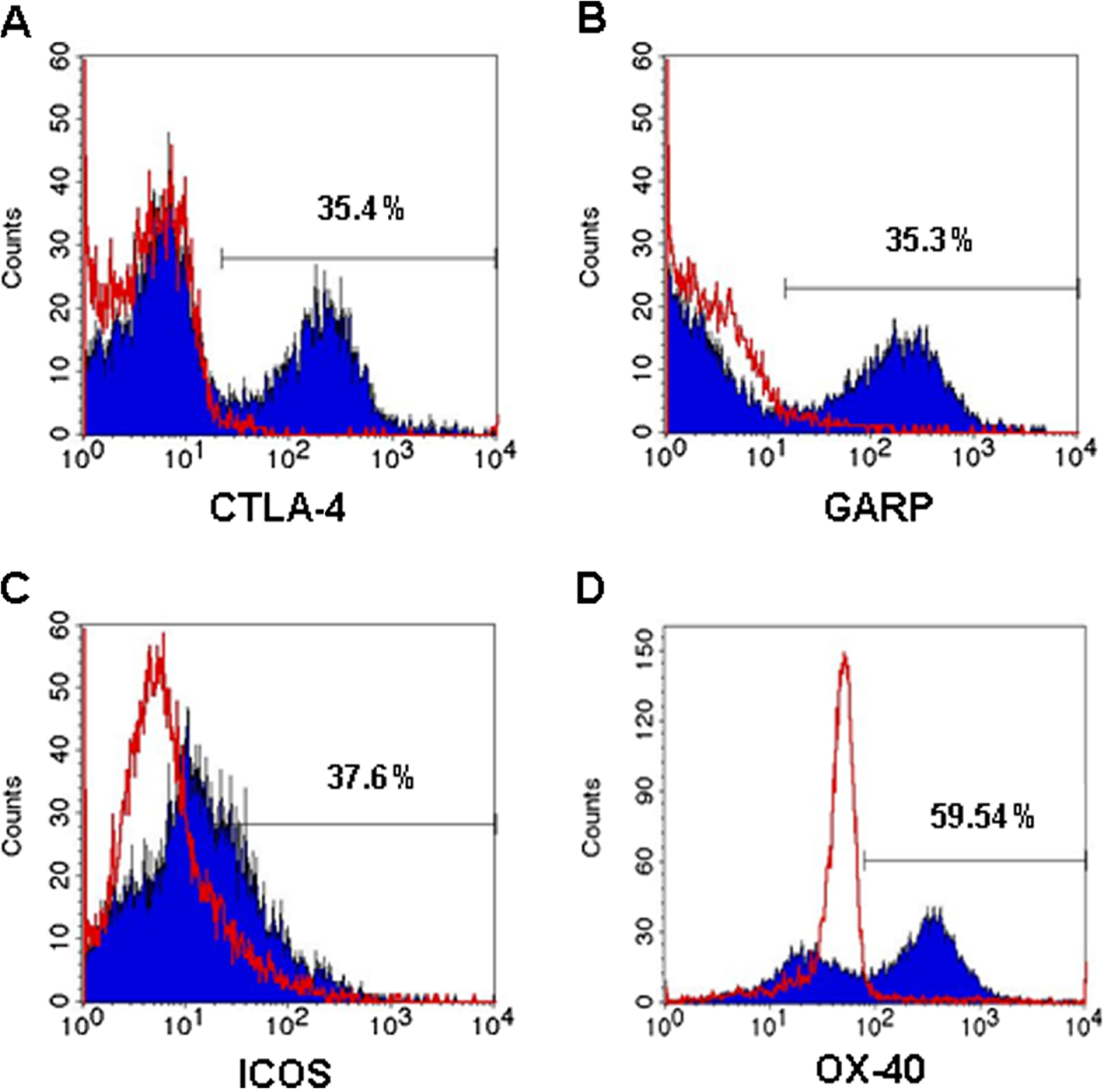
**CD4**^**+**^**CD25**^**-**^**cells up**-**regulate markers associated with regulatory phenotype.** TregPMA cells express the markers CTLA-4 (**A**), GARP (**B**), ICOS (**C**) and OX40 (**D**) associated with a regulatory phenotype and function. Representative histogram overlays are shown. In red CD4^+^CD25^-^ T cells at time 0 and in blue TregPMA cells after following PMA/ionomycin/anti-CD3 activation and 5 days in culture.

Additionally, we analyzed the expression of Foxp3, CTLA-4, GARP, ICOS and OX-40 markers which are known to be associated with a T regulatory cell phenotype and function [18-22]. All tested markers were found to be up-regulated in ~30% of the CD4^+^CD25^+^ T cells after activation (a representative result is shown in Figure 1D, Figure 2A, B, C, D). 

Similarly to previously reported data with iTreg cells generated *in vitro*[23], the cellular level of Foxp3 expression in the cells activated with PMA/ionomycin/anti-CD3 appears to be low (see: Figure 1B, D).

We will refer to the cells activated with PMA/ionomycin/anti-CD3 activation (iTreg cell population) as ‘TregPMA’ cells.

### TregPMA cells ameliorate the disease phenotype in a colitis transfer mouse model

IBD was induced in C.B-17 SCID mice by transfer of CD45RB^high^ cell population from BALB/C mice (positive control group) and was prevented by co-transfer of CD45RB^low^ cell population (negative control group) [24-27].

The read-out parameters of the development and progression of the colitis in the transfer mouse model are body weight loss and inflammation of the intestinal tissue.

**Figure 3:**
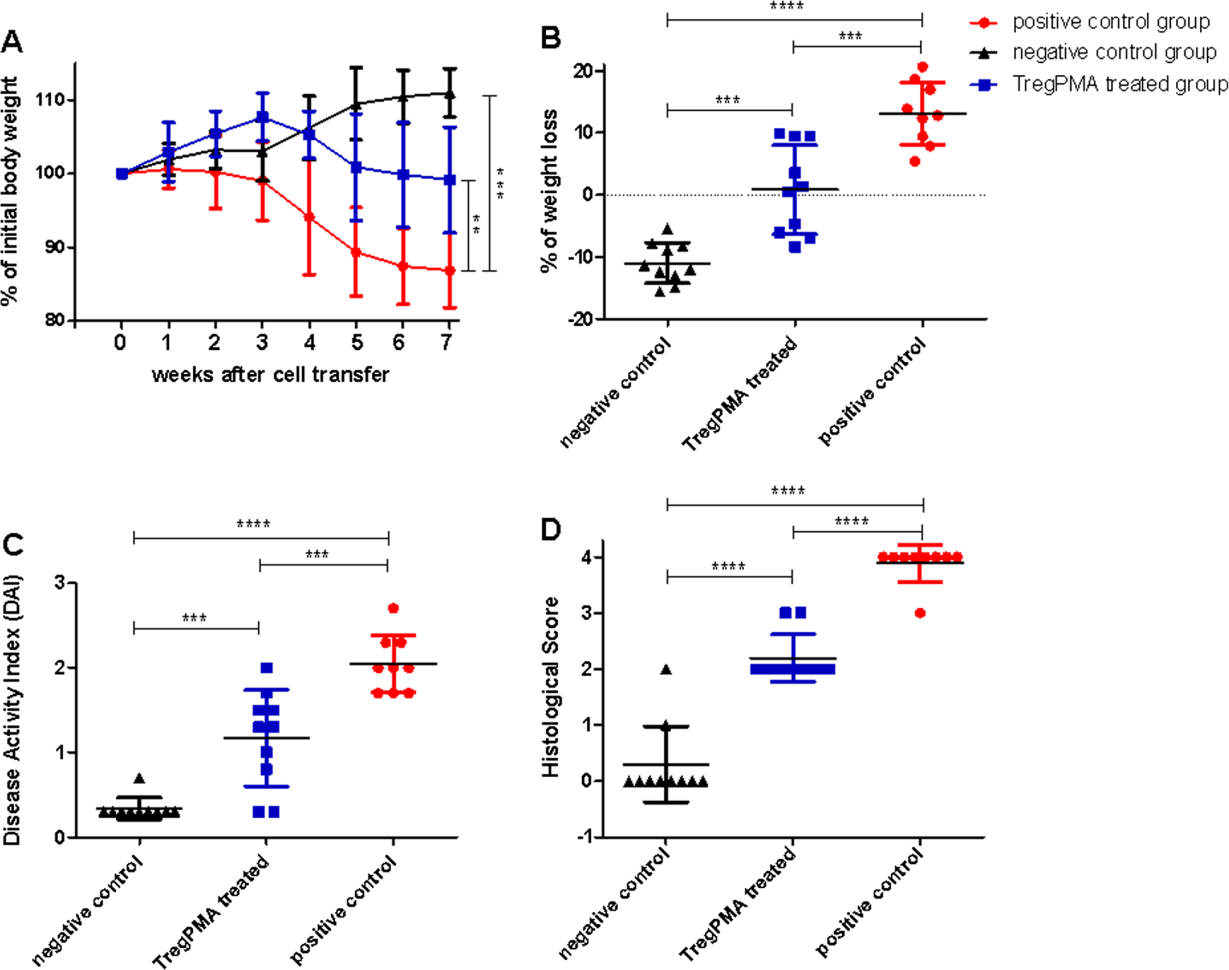
**Amelioration of the disease phenotype in TregPMA cell treated mice.** Mice that were injected with CD45RB^high^ cells only developed colitis (positive control group, n = 9) while the mice that were injected with CD45RB^high^ and CD45RB^low^ cells together remained healthy (negative control group, n = 10). The experimental group was injected with CD45RB^high^ and TregPMA cells (n = 10). Disease progression was assessed by changes in weekly body weight (**A**) as well as macroscopic and microscopic scores at day of sacrifice which comprises of Disease Activity Index (**C**) and Histological Score (**D**). TregPMA cell treated mice showed to have reduced weight loss over time (**A**) and at the time of sacrifice (**B**) when compared to positive control. The change of weight is expressed as the mean percentage of initial weight per group ± SD. Disease Activity Index (DAI), calculated from combining scores applied to weight loss, stool consistency at sacrifice and rectal bleeding divided by three, shows a significant difference between groups (**C**) as well as Histological Score of the H&E stained colon tissue sections (**D**). The data were analyzed using one-way ANOVA, followed by Bonferroni’s Multiple Comparison Test. *P < 0.05, **P < 0.01, ***P < 0.001, ****P < 0.0001.

The positive control group developed colitis and presented a relative decrease of initial body weight 86.88 ± 5.06% (n = 9) at sacrifice which was significantly lower than the negative control that reached 110 ± 3.29% (n = 10) of the initial body weight. The TregPMA cell treatment (injection of TregPMA cells together with CD45RB^high^ cells) reduced significantly the body weight loss to 99.18 ± 7.17% (n = 10) as compared to the positive control group (Figure 3A, B).

The Disease Activity Index (DAI), which is an indicator of colonic inflammation, reflecting weight loss, stool consistency and presence of rectal bleeding was determined at sacrifice. The DAI was significantly lower in mice treated with TregPMA cells (1.18 ± 0.55) when compared to the positive control group (2.04 ± 0.35), confirming the amelioration of the disease phenotype (Figure 3C).

Intestinal inflammation was further determined by histopathological analysis of colon tissue post mortem which includes evaluation of mononuclear and polymorphonuclear cell infiltrate, goblet cell depletion, loss of crypts, epithelial hyperplasia and manifestations of ulcerations and abscesses [24-27].

**Figure 4:**
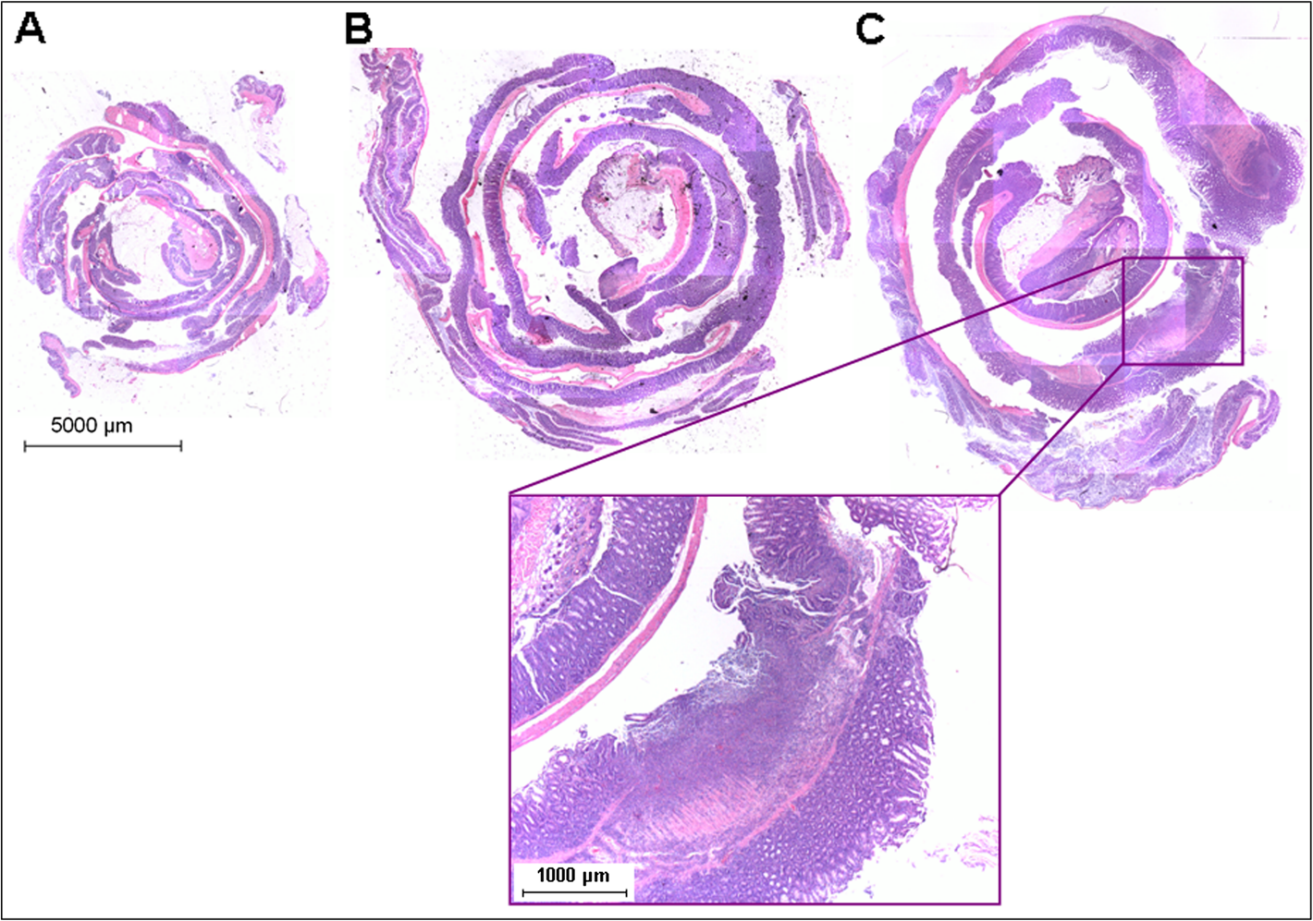
**Histopathology of mouse colon.** Representative whole-colon photographs of H&E stained tissue sections of negative control mouse (**A**), TregPMA cell treated mouse (**B**) and positive control mouse with ulceration manifestation (**C**).

A significant reduction of inflammation was observed in the colon tissue of the TregPMA cell treated mice when compared to the positive control group (Figure 3D). The spread of intestinal inflammation was less extensive and no ulcerations were observed in the TregPMA cell group (Figure 4B) which was similar to the negative control group (Figure 4A) while in the diseased control group they were commonly present (Figure 4C).

### Proinflammatory cytokine production in colon and in plasma of TregPMA cell treated mice is reduced

We next examined the influence of TregPMA cell treatment on the production of pro-inflammatory cytokines IL-6, IL-1β and TNF-α that have been previously described to be increased in CD45RB transfer colitis mouse model [28,29]. The level of IL-10 and TGF-β that have been shown to have a regulatory effect in experimental colitis were also analyzed [30,31]. Additionally the level of IL-17 cytokine that have been described to have both pro- and anti-inflammatory effect in IBD was measured [30,32-35].

**Figure 5:**
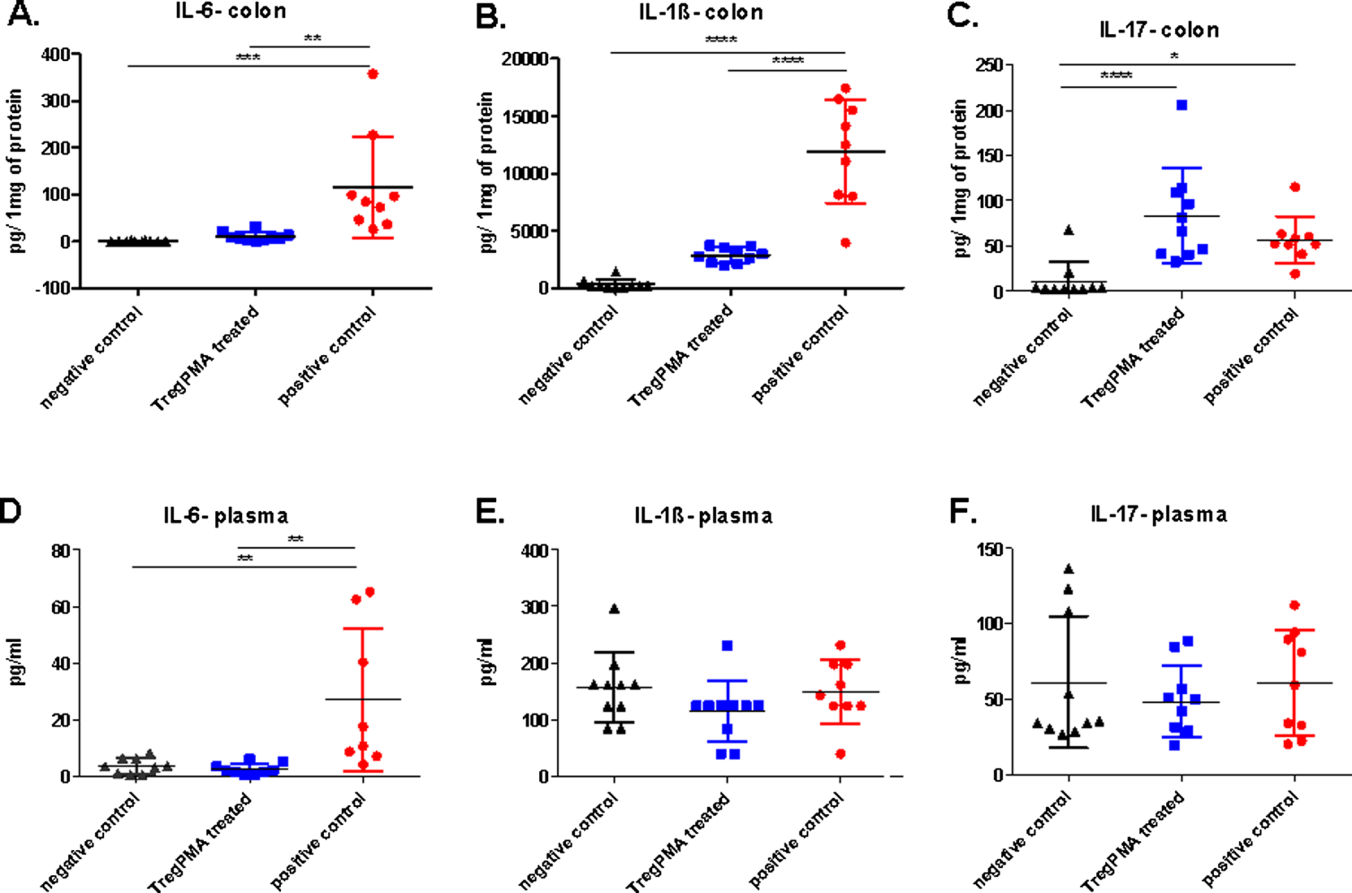
**Cytokine levels in mouse colon homogenates and plasma.** Colonic tissue homogenates and plasma levels of IL-1β (**A**, **D**), IL-6 (**B**, **E**) and IL-17 (**C**, **F**) of negative control group (n = 10), TregPMA cell treated group (n = 10) and positive control group (n = 9) were analyzed using a mouse cytokine magnetic bead-based multiplex assay. TregPMA cell treatment has shown to suppress increased levels of colonic IL-1β (**A**), IL-6 (**B**) and plasma IL-6 (**E**) in CD4^+^CD45RB^high^ CD25^-^ cell transferred colitic mice. The data were analyzed using one-way ANOVA, followed by Bonferroni’s Multiple Comparison Test. *P < 0.05, **P < 0.01, ***P < 0.001, ****P < 0.000.

The level of IL-6 was found to be decreased in TregPMA cell treated group in both colon homogenates and plasma at the day of sacrifice when compared to the positive control group (Figure 5A, D). IL-1β level was also found to be reduced after TregPMA cell treatment in colon homogenates but this decrease was not reflected in plasma (Figure 5B, E).

Elevated levels of IL-17 were found in colon tissue homogenates of TregPMA cell treated mice as well as in positive control group. However, the TregPMA cell treated group had higher level of colonic IL-17 than the positive control (Figure 5C). No significant difference between groups was observed in the levels of IL-17 expression in plasma (Figure 5F).

TNF-α, IL-10 and TGF-β levels in both colon homogenates and plasma showed no significant differences between groups (data not shown).

## Discussion

In this article we describe the generation of iTreg cells (TregPMA cells) *in vitro* from CD4^+^CD25^-^ cells by PMA/ionomycin/anti-CD3 activation based method. Several other groups have explored different strategies to generate iTreg from naive T cells *in vitro*, including use of TGF-β, all-trans-retinoic acid and the recently discovered regulatory cytokine IL-35 [36,37]. However, all these methods are more or less impeded with their own limitations [16]. Therefore, there is need for improvement in this field and our group designed and explored a new method of *in vitro* iTreg cells (TregPMA) generation which we report in this manuscript.

Mechanistically, PMA/ionomycin provides a potent stimulation allowing us to bypass the T cell receptor activation essential for Treg cell development and it prevents the emergence of CD4^-^CD8^+^ cells in the culture [17]. PMA activates protein kinase C [38] while ionomycin is a Ca^2+^ mobilizing agent [39]. This combination has been shown to up-regulate CD25 on T lymphocytes [17] and a high CD25 expression is a marker of the Treg cell phenotype [40]. Additionally, it was demonstrated that Ca^2+^ signaling is required for the development and function of Treg cells [41,42]. Therefore we expected that raising the intracellular levels of Ca^2+^ in naive T cells using ionomycin would have a synergistic effect with PMA in starting a regulatory developmental program in CD4^+^CD25^-^ cells. Additionally a low dose of IL-2 was used in cell culture since IL-2 is important for Treg cell homeostasis and maintaining their suppressive survival program [43] while anti-CD3 which is also present in the cell culture has been shown to maintain and expand Treg cells [44], however use of only anti-CD3 and IL-2 for *in vitro* CD4^+^CD25^-^ cell stimulation resulted in low cell viability and poor regulatory markers expression which indicates that additional stimulation with PMA/ionomycin is needed.

The TregPMA cells up-regulate CD25, and ~30% of the CD4^+^CD25^+^ T cells up-regulate CTLA-4, GARP, ICOS, OX-40 and Foxp3 which are important markers associated with regulatory cell phenotype and function [18-22,45,46]. Interestingly, the level of Foxp3 expression in TregPMA cells is quite low. Although Foxp3 expression is considered to be a main characteristic of Treg cells, Treg cells are recognized to be a heterogeneous cell population. Especially, it has been reported that CD4^+^CD25^+^ Foxp3^-^ cell population also can present regulatory function *in vitro* and *in vivo*[23].

The functionality of the TregPMA cells has been demonstrated *in vivo* by their potential to ameliorate the disease phenotype in a CD45RB transfer colitis mouse model. It cannot be fully excluded that the amelioration of the disease phenotype observed in the group of mice treated with the TregPMA cells does not partially involve cell types, other than regulatory, present in the cell suspension injected. Indeed, high numbers of CD45RB^high^ cells (6x10^6^/mouse) have been shown to reduce the severity of the disease in a mouse model of colitis [47]. However, in our study, the total number of cells injected (1,2x10^6^/mouse) was significantly lower and the amelioration of the severity of the disease was observed in all of the treated animals (n = 10) against 3 out of 5 in the mentioned study. All together, these data suggest that the observed protective effect is mostly related to TregPMA cells.

The disease amelioration after TregPMA treatment was accompanied by a decrease of the pro-inflammatory IL-1 β levels in the colon tissue which reflect the severity of experimental colitis [28]. Such a decrease in IL-1β was previously reported by Hirano and Kudo after treatment of CD45RB transfer colitis mouse model with both dexamethasone and anti-tumor necrosis factor-α (anti-TNF-α).

IL-1β stimulates the production of the pro-inflammatory cytokine IL-6 [48] which has been described to be up-regulated in mouse model of IBD [49] and in human ulcerative colitis or Crohn’s disease patients [50]. Simultaneously with the decrease of IL-1β in our study, IL-6 levels were found to be reduced in both colonic homogenates and plasma samples after treatment with TregPMA cells.

The concentration of IL-17 was found to be more elevated in colon homogenates of TregPMA cell treated mice than in the positive control group. Although the role of this cytokine in the initiation or pathogenesis of IBD is controversial, a protective function of IL-17 in the gut of CD45RB transfer colitis model has been previously noted [30,33,51]. Several groups have reported that both IL-17−/− CD45RB^high^ and IL-17R−/− CD45RB^high^ cells induce a more aggressive disease phenotype than wild type CD45RB^high^ cells [30,51]. Accordingly, our results show that an increased IL-17 level also correlated with less severe intestinal inflammation and lack of ulcerations.

IL-10 and TGF-β cytokine level in both mouse plasma and colon homogenates show no significant differences between experimental groups at the time of experiment termination (week 7). However, it does not exclude the possible influence of those cytokines on prevention of the colitis induction at earlier time point.

The cells that have been found mostly responsible for pathogenesis of CD45RB transfer model of IBD are Th1 cells. Recently it has been shown that IL-17 blocks the Th1 cell development *in vivo* via IL-17R on naive CD4^+^ T cells [30,51]. In the TregPMA cell treated group we also observe elevated level of IL-17 which correlates with disease amelioration. IL-17 can be produced by many types of cells among which are also T regulatory cells [52]. Therefore we are hypothesizing that *in vivo* disease amelioration by TregPMA cells is IL-17 mediated. As follow-up to this study, physiological relevance of TregPMA cells and their exact molecular mechanism of action needs to be determined.

## Conclusions

Induced regulatory T cells are promising tools for therapeutic applications in IBD [53] as well as in other autoimmune disorders and are of great scientific interest. Many techniques to generate inducible Treg cells from naive T cells have been developed and described [8,36,37,54,55]. Different activation methods to generate iTreg cells result in iTreg cells that are heterogeneous in phenotype. The *in vitro* PMA/ionomycin/anti-CD3 activation of CD4^+^CD25^-^ T cells has proven to generate functional iTreg cells (TregPMA cells) but the *in vivo* physiological relevance of TregPMA cell signaling needs to be further investigated.

## Abbreviations

PMA: Phorbol myristate acetate; iTreg: Induced regulatory T cells; nTreg: Natural regulatory T cells; IBD: Inflammatory bowel diseases; SCID: Severe combined immunodeficiency; IL: Interleukin; DAI: Disease activity index; TNF-α: Tumor necrosis factor- alpha.

## Competing interests

Three of the authors are employees of uniQure (AM, HP and VF), SM currently works in the laboratory of uniQure and SJD is a board member of uniQure.

## Authors’ contributions

AM carried out experiments and analyzed the data. AM and VF performed literature searches and wrote the article. SM and AAV participated in the animal experiment, SLM performed histological analysis of colon section, AM, VF, SM, AAV, SLM, HP and SJD contributed conceptually to the work, reviewed the manuscript and assisted with the editing of the paper. All authors read and approved the final manuscript.

## Pre-publication history

The pre-publication history for this paper can be accessed here:

http://www.biomedcentral.com/1471-230X/12/172/prepub
